# Editorial: Healthy Aging and the Community Environment

**DOI:** 10.3389/fpubh.2021.737955

**Published:** 2021-10-15

**Authors:** Chanam Lee, Xuemei Zhu, Anna Patricia Lane, Erja Portegijs

**Affiliations:** ^1^Department of Landscape Architecture and Urban Planning, Texas A&M University, College Station, TX, United States; ^2^Department of Architecture, Texas A&M University, College Station, TX, United States; ^3^Lee Kuan Yew Centre for Innovative Cities, Singapore University of Technology and Design, Singapore, Singapore; ^4^Faculty of Sport and Health Sciences and Gerontology Research Center, University of Jyväskylä, Jyväskylä, Finland

**Keywords:** active aging, aging-friendly community, age-friendly community, aging in place, community environment, healthy aging

Population aging is a global issue that brings many challenges and opportunities to modern societies. Healthy aging and age-friendly communities are important public health priorities which rely heavily on having supportive community environments that meet the needs of older adults. Such environmental supports may include barrier-free home and neighborhood environments, daily destinations located within an easy walking distance from home, connected and well-maintained sidewalks, and benches and good lighting along streets/paths, among many others. Requirements and preferences for environmental supports vary by different groups and generations of older adults. Healthy aging also requires supportive social and technological environments. Technology is an increasingly important factor driving changes in senior living. It offers a variety of solutions to support older adults' independence and social connectedness, as well as strategies to improve measurement approaches to better understand spatio-behavioral patterns of older adults' daily living.

The 15 articles included in this Special Issue respond to some of the critical needs presented by the current generation of older populations living in diverse socio-cultural contexts. They address five main themes central to meeting these needs, including (a) active living/aging, (b) health-related outcomes linked with community environments, (c) housing environments, (d) technological innovations and novel applications, and (e) methodological approaches. Collectively, these articles illustrate the potential of using interventions in housing, community, and technology environments to create aging-friendly communities that can bring beneficial outcomes for healthy aging and aging in place in both the short-term and long-term, as illustrated through a logic model in Figure 1 (1).

**Figure 1:**
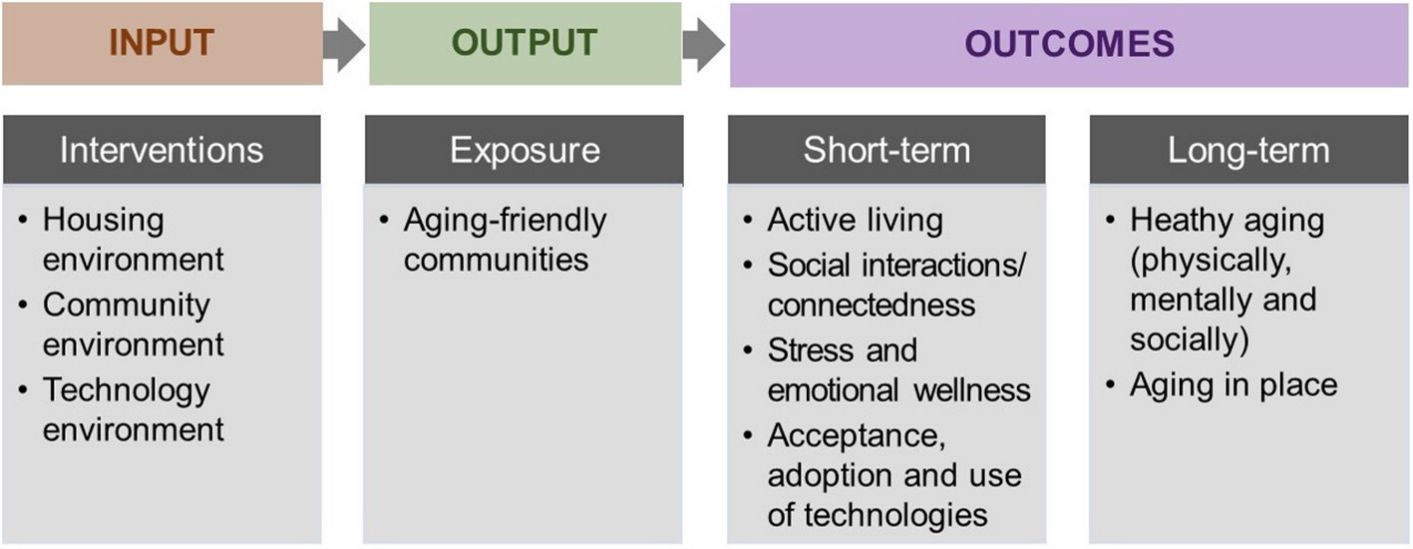
Logic model for promoting healthy aging through environmental interventions, represented by the papers included in this Special Issue.

First, five articles in this Special Issue address active living/aging, and examine how community environments can help older adults adopt and maintain active lifestyles. Two articles address the link between neighborhood environments and physical activity. Keskinen et al. studied community-dwelling older adults in Finland and showed the importance of proximate environments (e.g., diverse natural/green areas, street intersection density, residential density) in supporting older adults' moderate-to-vigorous physical activity (MVPA). They also demonstrated that the environment-MVPA associations may vary depending on the days of the week (i.e., weekdays vs. weekends). Another Finnish study by Portegijs et al. demonstrated that barriers near (≤500 m) home were negatively associated with physical activity among community-dwelling older adults, while attractive destinations further away (>500 m) were positive correlates. He et al. conducted their study in a highly dense Chinese megacity, exploring the relationship between built environments and walking among older adults. Their results revealed limited roles of neighborhood walkability (street connectivity being the only significant factor) in leisure-time walking. This is somewhat different from another study in this issue (Herbolsheimer et al.) and the previous literature that reported significant results for multiple walkability measures and strong relationships between built environments and transportation walking among seniors (2, 3). Two other studies under this theme were carried out in North American cities and identified specific features/elements of the neighborhood environment associated with transportation walking among older adults. Those features include density, crosswalks, and parks or outdoor fitness amenities in Herbolsheimer et al.; and crosswalks, pedestrian signals, unattended dogs, lighting at night, religious institutions, and slope in the study by Lee et al. These studies together highlighted diverse community factors associated with walking and overall physical activity among older adults.

The second theme in this Special Issue is represented by three articles linking older adults' health-related outcomes with walking and the community environment in the U.S. Zhong et al. reported that walkability-related features of the neighborhood, such as transportation infrastructure, land uses, land covers, population densities, and development activities, were associated with social interactions, including intergenerational activities, among older adults from Austin, Texas. Roe et al. found that walking in an urban green district (a quiet residential area with front gardens and street trees) brought positive changes in emotional well-being and stress physiology for senior residents. A national cohort study by Jones et al. detected a negative relationship between neighborhood Walk Score and the incident hypertension risk in black and white older adults. In addition to the behavioral outcomes discussed under the first theme of active living/aging, studies in this second theme add examples of physical, mental, and social health outcomes tied with community environments.

Third, three studies carried out in Australia, Finland, and Ghana address the roles of housing environments in diverse sociocultural contexts. The Australian study (Byles et al.) explored the relationship between housing types and the care needs among 12,432 women. Compared to those living in a house, older women living in an apartment and a retirement village/hostel had moved faster to a residential aged care facility. The Finnish study (Jolanki) used a qualitative approach to interview 36 residents of a communal senior housing complex. The results suggested that the design intention of providing accessible, safe, and affordable environments was met, while differences existed among the residents in terms of the specific environmental features and characteristics important to them. Another qualitative study (Alaazi et al.) involving older adults residing in slum and non-slum neighborhoods in Ghana found similar environmental barriers (e.g., poor drainage, lack of sidewalks, poor housing conditions, unsanitary conditions) to health in both types of neighborhoods. They also pointed out challenges in developing effective policy interventions to support living environments that are affordable, safe, and accessible for older adults. These studies on housing environments contribute to the discussions on the need to consider the community environment at multiple spatial scales, ranging from housing and immediate surroundings to the neighborhood and the larger city or socio-cultural contexts.

Fourth, this issue considered the emergence of technological innovations and novel applications to support healthy aging. Two articles address this topic. A U.S. study by Seo et al. introduced a novel art–technology intergenerational community program designed to support older adults' health, well-being, and intergenerational connectedness. The 18 participants of this program reported benefits in their relationships with student volunteers and with their own family members. den Haan et al. from the Netherlands and Australia presented a living lab approach to show how a social learning environment can be created to facilitate the acceptance, adoption, and sustainable use of smartphone technologies. They highlighted the promise of using super-users, who are previously trained peer users, to create supportive peer learning mechanisms and peer support environments. These articles on technologies offer valuable insights on the new or complementary approaches to empower older adults to remain engaged and age in place.

In addition, papers in this Special Issue employ a variety of measurement methods, ranging from the traditional self-report methods such as surveys, interviews, and focus groups, to objective measures using geographic information systems, accelerometers, street/environmental audits, noise and air quality sensors, and smartwatches. Two articles specifically focus on methodological approaches to facilitate healthy aging research. A literature review article by Zanwar et al. provided a synthesis of the growing body of interdisciplinary studies and methods on connected technologies such as wearable and embedded sensors and processors that connect people with their environments. Lin et al. validated the global self-rated health and happiness measures for use in community-dwelling older adults using a large sample in Taiwan, with the age- and gender-specific scoring systems. These studies point to the need for valid measurement methods tailored for older adults and the challenges in using technology-based methods in studies involving or about older adults due to reasons such as their health conditions and low acceptance levels.

In summary, articles in this Special Issue addressed the role of community environments in healthy aging, which encompassed diverse scales (e.g., homes, streets, neighborhoods) and domains of the built, social, and technological environment. They were carried out in multiple socio-cultural contexts from eight countries (Australia, Canada, China, Finland, Ghana, the Netherlands, Taiwan, and the U.S.) and five continents (Africa, Asia, Australia, Europe, and North America). Manuscript types are diverse, including one literature review article, four qualitative studies, one validation study, one pilot study, and eight quantitative studies. Most studies included in this issue are cross-sectional, and longitudinal and intervention studies are not well-represented. There is a need for continued work in this area addressing the full range of built, social, and technological environments and their causal impacts on healthy aging outcomes.

Collective findings from these studies offer valuable knowledge for future research and practice in terms of facilitating healthy aging and aging in place through supportive environments. This Special Issue highlights some of the promising efforts that interdisciplinary scholars have been making toward consolidating the link between community/technological environments and healthy aging.

## Author Contributions

CL drafted and finalized the editorial. XZ developed the figure and reviewed/edited the editorial. AL and EP reviewed/edited the editorial. All authors contributed to the article and approved the submitted version.

## Conflict of Interest

The authors declare that the research was conducted in the absence of any commercial or financial relationships that could be construed as a potential conflict of interest.

## Publisher's Note

All claims expressed in this article are solely those of the authors and do not necessarily represent those of their affiliated organizations, or those of the publisher, the editors and the reviewers. Any product that may be evaluated in this article, or claim that may be made by its manufacturer, is not guaranteed or endorsed by the publisher.
